# Genotype-by-environment and QTL-by-environment interactions in sweet cherry (*Prunus avium* L.) for flowering date

**DOI:** 10.3389/fpls.2023.1142974

**Published:** 2023-03-02

**Authors:** Camille Branchereau, Craig Hardner, Elisabeth Dirlewanger, Bénédicte Wenden, Loïck Le Dantec, David Alletru, Julien Parmentier, Anton Ivančič, Daniela Giovannini, Federica Brandi, Gregorio Lopez-Ortega, Federico Garcia-Montiel, Bénédicte Quilot-Turion, José Quero-García

**Affiliations:** ^1^ INRAE, Univ. Bordeaux, Unité Mixte de Recherche Biologie du Fruit et Pathologie (UMR BFP), Villenave d’Ornon, France; ^2^ Queensland Alliance for Agriculture and Food Innovation, The University of Queensland, Brisbane, QLD, Australia; ^3^ INRAE, Unité Expérimentale (UE) 0393, Unité Expérimentale Arboricole, Toulenne, France; ^4^ Faculty of Agriculture and Life Sciences, University of Maribor, Hoce, Slovenia; ^5^ Consiglio per la ricerca in agricoltura e l'analisi dell'economia agraria (CREA), Research Centre for Olive, Fruit and Citrus Crops, Forli, Italy; ^6^ Atlantic Green, Ctra. Almonde El Rocio, Huelva, Spain; ^7^ Instituto Murciano de Investigación y Desarrollo Agrario y Alimentario (IMIDA), Instituto Murciano de Investigación, y Desarrollo Agrario y Alimentario, Murcia, Spain; ^8^ INRAE, Unité de Recherche (UR) 1052 GAFL, Montfavet, France

**Keywords:** sweet cherry, flowering, genotype-by-environment interactions, QTL, QTL-by-environment interactions

## Abstract

In sweet cherry (*Prunus avium* L.), flowering date is strongly dependent on the environment conditions and, therefore, is a trait of major interest for adaptation to climate change. Such trait can be influenced by genotype-by-environment interaction (G×E), that refers to differences in the response of genotypes to different environments. If not taken into account, G×E can reduce selection accuracy and overall genetic gain. However, little is known about G×E in fruit tree species. Flowering date is a highly heritable and polygenic trait for which many quantitative trait loci (QTLs) have been identified. As for the overall genetic performance, differential expression of QTLs in response to environment (QTL-by-environment interaction, QTL×E) can occur. The present study is based on the analysis of a multi-environment trial (MET) suitable for the study of G×E and QTL×E in sweet cherry. It consists of a sweet cherry F_1_ full-sib family (n = 121) derived from the cross between cultivars ‘Regina’ and ‘Lapins’ and planted in two copies in five locations across four European countries (France, Italy, Slovenia and Spain) covering a large range of climatic conditions. The aim of this work was to study the effect of the environment on flowering date and estimate G×E, to carry QTL detection in different environments in order to study the QTL stability across environments and to estimate QTL×E. A strong effect of the environment on flowering date and its genetic control was highlighted. Two large-effect and environment-specific QTLs with significant QTL×E were identified on linkage groups (LGs) 1 and 4. This work gives new insights into the effect of the environment on a trait of main importance in one of the most economically important fruit crops in temperate regions. Moreover, molecular markers were developed for flowering date and a strategy consisting in using specific markers for warm or cold regions was proposed to optimize marker-assisted selection (MAS) in sweet cherry breeding programs.

## 1 Introduction

The phenotype of an individual is the result of a combination of the effect of its genotype, the external environment, and the interaction between the genotype and environmental variations (Genotype-by-environment interactions, G×E). G×E is a common phenomenon referring to differences in the response of genotypes to different environments (Allard and Bradshaw, 1964; Falconer and Mackay, 1996). The presence of G×E can affect the genetic advance obtained from selection (i.e. superior cultivars in one environment are not necessarily superior in another environment) and therefore is a major concern for plant breeders. Two major sources of G×E exist: (1) rank-change interaction (or crossover interaction), when genotypes are ranked in different orders in different environments; and (2) scale-change interaction (or level-of-expression interaction), when genotypic differences vary across environments (heterogeneity of genetic variances across environments). Conventionally, multi-environment trials (METs) are conducted to assess the performance of a common set of genotypes in different environments and evaluate G×E (Smith et al., 2005). Many statistical methods have been developed for a precise description of G×E and nowadays, linear mixed models where genotypes are treated as random effect factors are frequently used (Smith et al., 2005; Malosetti et al., 2013; van Eeuwijk et al., 2016).

In perennial plant species, G×E has been mostly studied in forest tree species for traits related to tree height and trunk diameter (Li et al., 2017; Bird et al., 2022). More recently, horticultural fruit tree species such as apple (Jung et al., 2020), macadamia (Hardner, 2017), sweet cherry (Hardner et al., 2019) and peach (Hardner et al., 2022) have also been studied. For instance, in sweet cherry, low additive G×E and high additive genomic correlations among environments were estimated for maturity date in a germplasm of 597 cultivars, accessions and unselected offspring planted in three locations in Europe and one location in the USA (Hardner et al., 2019). In apple, genomic G×E explained 18 and 12% of the phenotypic variance of floral emergence and harvest date in a population of 534 genotypes planted across six European countries (Jung et al., 2020). In summary, in these horticultural crops, several traits related to phenology, yield and fruit quality (e.g. sweetness) have been studied. Nevertheless, little is known about the environmental stability of genetic effects for flowering time.

In the current global warming context, flowering date (FD) is a trait of major interest in temperate fruit tree species such as sweet cherry (*Prunus avium* L.). FD is highly dependent on the climatic conditions, therefore, breeding programs tend to develop early and late blooming cultivars according to their area of production (Quero-García et al., 2017). Early flowering cultivars are promoted in warm regions to avoid high temperatures during flowering while late cultivars are best suited for cold areas to avoid spring frost damages. Moreover, FD is dependent on the dormancy period in which temperate fruit trees enter during winter to stop meristem activity and prevent frost damages (Lang et al., 1987). The length of this period varies according to climate conditions and individuals, as specific amounts of chill and heat, known as ‘chilling requirements’ (CRs) and ‘heat requirements’ (HRs), are required to release dormancy (Alburquerque et al., 2008). However, nowadays, few low-chilling varieties (with CRs varying from 300 to 800 chilling hours) are available (Wenden et al., 2017). This is even more problematic in the context of global warming, with the increase of temperatures in autumn and winter, which has already provoked serious production losses on cultivars with high CR (Quero-Garcia, comm. pers.). Furthermore, increases in spring temperatures with global warming have entailed a significant advance of flowering dates of cherry cultivars in numerous production areas (Luedeling, 2012; Wenden et al., 2017), with a subsequent increase in risk of frost damage. Although some work has been conducted in several breeding programs to investigate tolerance or resistance to cold, and hence to frost damage, very few cultivars carrying these favorable traits have been released so far and none has reached commercial importance (Quero-García et al., 2017; Wenden et al., 2017). To date, knowledge about G×E and the stability of whole genome effect for FD in sweet cherry is missing.

FD is a quantitative trait with high broad sense heritability and genetic approaches have led to the identification of many FD QTLs, highlighting a complex genetic control. Due to the high genomic synteny within the *Prunus* genus, FD QTLs have been identified in sweet cherry and other species in similar chromosomal regions (Dirlewanger et al., 2004; Dirlewanger et al., 2012; Castède et al., 2014; Sánchez-Pérez et al., 2014; Calle et al., 2020). Although QTLs have been detected on all linkage groups (LGs), the two largest-effect loci were located on LGs 1 and 4 (Fan et al., 2010; Dirlewanger et al., 2012; Castède et al., 2014; Sánchez-Pérez et al., 2014; Cai et al., 2018; Calle et al., 2020; Branchereau et al., 2022). Castède et al. (2014) dissected sweet cherry FD into CRs and HRs and detected QTLs for FD, CRs and HRs co-localizing in the LG4 region. The QTL on LG1 covers the genomic region of the well-known *DORMANCY-ASSOCIATED MADS-box* (*DAM*) genes (Bielenberg et al., 2008; Castède et al., 2015; Calle et al., 2021). Recently, candidate genes involved in auxin responses and splicing have been identified in the QTL on LG4 using the ‘Regina’ sweet cherry genome sequence and transcriptomic analyses (Branchereau et al., 2022).

In sweet cherry, little is known about the stability of QTL effects for FD across environments. As the overall genetic performance, QTLs can be expressed differently in different environments: a QTL can be significant for a given trait in one environment but not in another. This is called QTL-by-environment interaction (QTL×E) and a QTL with large QTL×E interaction is less stable than a QTL with small QTL×E. In *Prunus*, Asíns et al. (1994) were the first to use QTL detections as an approach to study G×E. Authors studied the effect of years on QTL detection for several quantitative traits in almond. They highlighted that only few QTLs behaved homogeneously over the years and showed that the presence of G×E was the most likely cause of this phenomenon.

The presence of G×E is a main concern for breeders as it complicates the identification of superior cultivars and reduces gains from selection. The identification of stable genotypes (low G×E) over a wide range of environments is of main importance, especially in locations associated with strong environmental fluctuations. Moreover, information about QTL×E is essential to develop marker-assisted selection (MAS) for FD according to the area of production.

The main objectives of this study were to (i) estimate G×E in a sweet cherry MET, (ii) perform QTL detection in order to study the QTL stability over environments, and (iii) evaluate QTL×E interactions for FD in sweet cherry. This work should contribute to increase the efficiency of sweet cherry breeding programs.

## 2 Material and methods

### 2.1 Plant material

**Figure 1:**
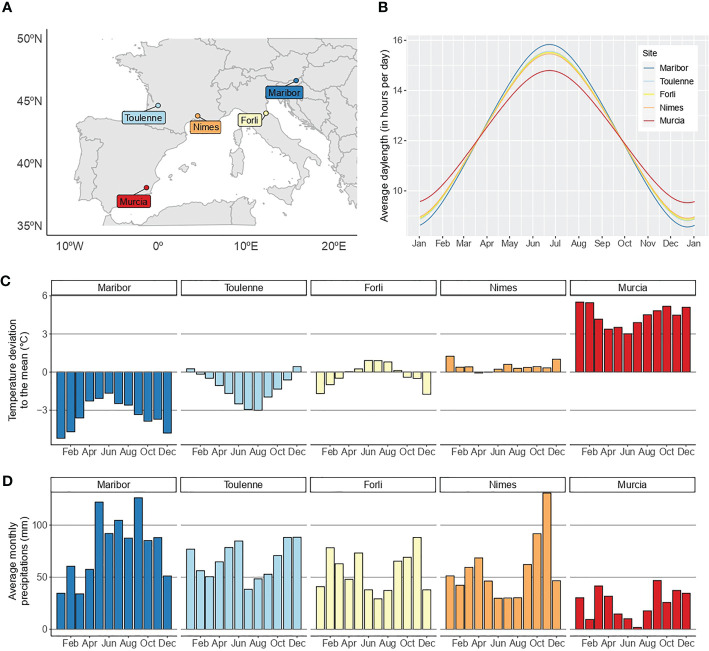
Location and environment characterization of the five experimental sites across Europe. **(A)**, location of the five sites in Europe. **(B)**, average day length in the five sites. **(C)**, temperature deviation to the overall monthly mean. **(D)**, average monthly precipitations in the five sites. In **(B–D)**, the climatic data used is from 2010 to 2021. A color scale from blue to red was chosen to represent the sites according to the temperature data.

An F_1_ sweet cherry full-sib family derived from the cross between ‘Regina’ and ‘Lapins’ cultivars was used for this study. ‘Regina’ is a late blooming German cultivar, whereas ‘Lapins’ is an early-intermediate blooming cultivar from Canada. This family, hereafter called R×L, consists of clones of 121 hybrids planted in a MET in five locations across four European countries: Forli (north-eastern Italy), Maribor (north-eastern Slovenia), Murcia (south-eastern Spain), Nimes (south-eastern France) and Toulenne (south-western France) (**Table S1**; **Figure 1A**). Daylenght is similar in the different locations (**Figure 1B**) while temperatures and precipitations are highly contrasted (**Figures 1C, D**). In Maribor, the climate is continental with cold winters and quite warm summers. Important rain precipitations are observed all year long. It is highly contrasted to Murcia, which is dry year-round and has mild winters and very hot summers. Climates in Forli, Toulenne and Nimes are intermediate (**Figures 1B–D**). Orchards were irrigated in all locations except Maribor and Toulenne. Trees were planted every 2.5 to three meters in rows separated by five meters. G×E studies require replication of genetic effects across MET, therefore, the 121 R×L genotypes were grafted (clonally replicated) in two copies in all environments (rootstocks: Maxma Delbard^®^ 14 or Colt) and planted in a random design. However, genotypes are not all present in the five locations and the number of replicates per genotype varies between sites (**Table S2**).

### 2.2 Flowering date phenotyping

Two FD stages were scored in all locations: beginning of flowering (BF), when approximately 10% of the floral buds reached full bloom, and full flowering (FF), when 75% of the floral buds reached full bloom. Trees were observed from three to four times a week during the season to score the different flowering stages in Julian days (JDs). FD was assessed at each location for several seasons: FD was scored in 2018, 2019, 2020 and 2021 in Forli; in 2017, 2019 and 2021 in Maribor; in 2017, 2018, 2019 and 2020 in Murcia; in 2017, 2018, 2019 and 2021 in Nimes; and in 2016, 2017, 2018, 2019 and 2021 in Toulenne. ‘Environments’ were considered as the combination of the location and the year (season) of the trial. Therefore, the MET consisted of twenty unique location **×** season environments.

### 2.3 Environment characterization

As temperature is the most important climatic factor for flowering in perennials like sweet cherry, the twenty environments were characterized using temperature data from October to May, a period covering dormancy (endodormancy and ecodormancy) and flowering. In each location separately, from October to May, a daily mean temperature was calculated using temperature data from 2010 to 2021. Then, the temperature data from each season we studied was represented as a deviation to the overall mean. Plots are available in **Figures S1**–**S5**, for Forli, Maribor, Murcia, Nimes and Toulenne, respectively. This type of representation allows to visualize in each location when the temperatures were either lower or higher than the mean.

### 2.4 Flowering date distribution, correlations and heritabilities

#### 2.4.1 Phenotypic data review

Distribution, mean, minimum and maximum values of BF and FF were estimated for each location-by-season environment. Additionally, Spearman correlation coefficients between years within each location and between locations for each year were calculated.

#### 2.4.2 Analyses of G×E

In order to describe the phenotype profile of the 121 R×L individuals across environments, norms of reaction were obtained with ‘ggplot2’ R package (Wickham, 2016). For each individual in each environment, the average phenotypic value was calculated from the replicates. Norms of reaction were obtained across locations in each year of study and across years in each location of study.

A G×E model was fitted to multi-trial data to estimate the genetic architecture of traits. As the MET is unbalanced (e.g. different number of replicates per genotype, some genotypes lacking in several environments, different years of measurement available in different locations), the mixed model approach was used. To reduce the heterogeneity of variance between environments and hence potential influence of heterogeneity of variance on G×E, FD observations within each environment were scaled by the raw phenotypic standard deviation of their respective environment (Hardner, 2017). Analyses were performed for BF and conducted in R using ASReml-R package version 4 (Butler et al., 2017).

The general model of the phenotype of an i^th^ individual in the k^th^ block at the l^th^ trial for the j^th^ season was


$$y_{ljki}=m+e_{lj}+eb_{ljk}+g_{i}+ge_{lji}+r_{ljki}$$

where

*m* was the general mean across all trials, blocks within trials, seasons at trials, and individuals

*e_lj_* was the fixed effect of the *lj^th^* environment (i.e. j^th^ season at the l^th^ trial)

*eb_ljk_* was the fixed effect of the k^th^ block at lj^th^ environment

*g_i_* was the average total (*i.e.*, additive + non-additive) genetic effect of the i^th^ individual across environments with distribution N(0,**G**g) where **G**g was the variance-covariance matrix among average total genetic effects across environments given as **G**g = I*v where **I** was the identity matrix of relationships among individuals and *vg* was the unknown total genetic variance across environments (where * was the Kronecker product)

*ge_lji_* was the random environment specific total genetic effect of the i^th^ individual at the lj^th^ environment with distribution N(0,**G**ge) where **G**ge was the variance-covariance matrix among environment specific total genetic effects at each of the lj^th^ environments given as ***G*** *ge* = I* ***V*** *ge* where **I** was the identity matrix of relationships among individuals, and **V**ge was the covariance matrix of environment specific total genetic effects among environments

*r_ljki_* was the residual effect for each phenotype observation with distribution N(0,**R**) where **R** was a block diagonal matrix of residual covariance among seasons for individuals at each trial, i.e. **V**r_l_ where **V**r_l_ was given as **I**_l_*Vr_l_ where **I**_l_ was an identity matrix among individuals at the l^th^ trial and **V**r_l_ was the residual covariance matrix among seasons at the l^th^ trial.

Parameters of the model (i.e. covariance components) were estimated using Restricted Maximum Likelihood implemented in the R package ASREML-R v4 (Butler et al., 2017). Tests of significance for fixed effects were done with Wald test (Kenward and Roger, 1997). The diagonal of the variance covariance matrix was referred to the interaction variance in each environment *lj*, *v_G_*_×_ *_E_* (*lj*), in other words, the variance in each environment that was not explained by the variance of the main effect of the genotype across environments (*v_G_*).

The total genetic variance in environment *lj* was *var*_*GEI*_(*lj*)=*v*_*G*_+*v*_*G*×*E*_(*lj*) . The total genetic covariance between environments *lj* and *l’j’* was *cov*_*GEI*_(*lj*,*l*'*j*')= *v*_*G*_+*cov*_*G*×*E*_(*lj*,*l*'*j*') , where *cov*_*G*×*E*_(*lj*,*l*'*j*') was the covariance from the variance-covariance matrix between environments *lj* and *l’j’*. Therefore, genetic correlation between environments *lj* and *l’j’* was estimated as


$$cor\left(lj,l'j'\right)=\;\frac{cov_{GEI}\left(lj,l'j'\right)}{\sqrt{var_{GEI}\left(lj\right)\;\times \;var_{GEI}\left(l'j'\right)}}$$

This correlation was used to estimate the magnitude of G×E due to ranking changes (when *cor*(*lj*,*l*'*j*') <1). Heatmaps of pair-wise genetic correlations were generated using the ‘ggplot2’ R package (Wickham, 2016).

#### 2.4.3 Heritabilities

Broad-sense heritability was estimated in each location from the analysis of variance based on the following mixed model:


$$y_{ijk}=µ+g_{i}+s_{j}+b_{k}+e$$

where y_ijk_ is the phenotypic value of the k^th^ replicate of the i^th^ individual in the j^th^ season, µ is the mean value of the trait, g_i_ is the random genotypic effect of individual i, s_j_ is the fixed effect of season j, b_k_ is the fixed effect of the block k (or replication), and e is the residual of the model. This linear mixed-effects model was fitted in R using the *lme4* package (Bates et al., 2015). Broad-sense heritability of individual location clonal means (H^2^) was then estimated as:


$$\;H^{2}=\frac{\sigma_{\text{g}}^{2}}{\sigma_{\text{g}}^{2}+\;\frac{\sigma_{\text{e}}^{2}}{nr}}\;\;$$

where $\sigma_{\text{g}}^{2}$ is the genetic variance, $\sigma_{\text{e}}^{2}$ the residual variance, n is the number of seasons and r is the number of replicates per genotype.

Broad-sense heritability of clonal means across the complete MET (called ‘MET broad-sense heritability’) was estimated with a pool analysis across environments (i.e. location × season) using the following mixed model:


$$y_{ijk}=µ+G_{i}+E_{j}+GE_{ij}+e$$

where y_ijk_ is the phenotypic value of the k^th^ replicate of the i^th^ individual in the j^th^ environment, µ is the mean value of the trait, G_i_ is the random genotypic effect of individual i, E_j_ is the fixed effect of the environment j, GE_ij_ is the random effect of the interaction between the i^th^ genotype and the j^th^ environment, and e is the error term. MET broad-sense heritability ( $H_{MET}^{2}$ ) was then estimated using the following equation:


$$H_{MET}^{2}=\;\frac{\sigma_{g}^{2}}{\sigma_{g}^{2}+\;\frac{\sigma_{ge}^{2}}{\text{e}}+\;\frac{\sigma_{e}^{2}}{\text{er}}}$$

where $\sigma_{g}^{2}$ , $\sigma_{ge}^{2}$ , and $\sigma_{e}^{2}$ are the genotypic, genotype-by-environment interaction, and error variance components, respectively, and e and r are the number of environments and of replicates within each environment, respectively.

Moreover, the fraction of phenotypic variation explained by the genotype, the environment and their interaction (G×E) was estimated from the mixed model fitted for the calculation of the MET heritability using the *insight* R package (Lüdecke et al., 2019).

### 2.5 QTL analyses

The R×L family was genotyped using single nucleotide polymorphism (SNP) markers from the RosBREED cherry 6K Illumina Infinium^®^ SNP array (Peace et al., 2012) and genetic maps have already been published (Klagges et al., 2013; Castède et al., 2014). QTL detection analyses were performed for BF and FF using the Multiple Interval Mapping (MIM) method implemented in MultiQTL V2.6 software (http://www.multiqtl.com). In each environment, the genotype means were used to perform QTL mapping. Analyses were carried out separately for ‘Regina’ and ‘Lapins’ parental maps by using the ‘single QTL model’ (i.e. one QTL per LG). For each location, both single-year and multi-year models were utilized. Moreover, a multi-location—multi-year analysis was performed. Both multi-year and multi-location—multi-year analyses were carried through the multi-environment model available in MultiQTL. In all analyses, QTL significance thresholds were determined by chromosome-wide permutation tests (1000 iterations) as described in Dirlewanger et al. (2012). A wide-genome type I error of 5% was chosen and used to calculate the type I error at the chromosome level as explained in Saintagne et al. (2004). When performing multi-environment analyses, a single QTL position (in cM) and a single LOD (logarithm of the odds ratio) value are given while values of percentage of variation explained (PVE) are estimated for each environment. For ease of reading, the mean PVE value across environments is presented.

### 2.6 Analysis of QTL×E interactions

QTL×E analyses were performed on a selection of QTLs that, in multi-location—multi-year analyses, explained the largest part of the phenotypic variation, had the highest LOD values, and were consistently significant for the two FD stages. For each QTL, we selected the two closest flanking markers and created, for each R×L hybrid, a variable containing the genotypes of the two markers (with the code AB for heterozygous and AA for homozygous).

In order to estimate the strength of the interaction for each QTL, a step-wise approach was undertaken. Firstly, we studied each QTL independently, in single-QTL models, in order to test the significance of the QTL main effect and the significance of the interaction.

Single QTL models are an extension of the G**×**E (or non-QTL) model where the total genetic effect of the i^th^ individual, g_i_, is decomposed into q_i_ and x_i_ where q_i_ is the QTL main effect and x_i_ is the effect of the background genotype. The general model was:


$$y_{ljki}=m+e_{lj}+eb_{ljk}+q_{i}+qe_{lji}+x_{i}+xe_{lji}+r_{ljki}$$

where

*m* was the general mean across all trials, blocks within trials, seasons at trials, and individuals

*e_lj_* was the fixed effect of the *lj^th^* environment (i.e. j^th^ season at the l^th^ trial)

eb_ljk_ was the fixed effect of the k^th^ block at lj^th^ environment

q_i_ was the fixed QTL main effect in the i^th^ individual

qe_lji_ was the fixed environment specific QTL effect of the i^th^ individual at the lj^th^ environment

x_i_ was the background genetic effect of the i^th^ individual with distribution N(0,**G**g) where **G**g was the variance-covariance matrix among total genetic effects given as ***G*** *g* = ***I*** *vg where **I** was the identity matrix of relationships among individuals and *vg* was the unknown total genetic variance (where * was the Kronecker product)

xe_lji_ was the random environment specific background genetic effect of the i^th^ individual at the lj^th^ environment with distribution N(0,**G**ge) where **G**ge was the variance-covariance matrix among environment specific total genetic effects at each of the lj^th^ environments given as ***G*** *ge* = ***I*** * ***V*** *ge* where **I** was the identity matrix of relationships among individuals, and **V**ge was the covariance matrix of environment specific total genetic effects among environments

r_ljki_ was the residual effect for each phenotype observation with distribution N(0,**R**) where **R** was a block diagonal matrix of residual covariance among seasons for individuals at each trial, i.e. **V**r_l_ where **V**r_l_ was given as **I**_l_*Vr_l_ where **I**_l_ was an identity matrix among individuals at the l^th^ trial and **V**r_l_ was the residual covariance matrix among seasons at the l^th^ trial.

In single-QTL models, the variance due to other QTLs is accounted for by the background genotype.

Then, we selected the QTLs that showed either a significant main effect (p-value< 0.05), a significant interaction effect, or both, and grouped them in a “complete” model to test whether these effects remained significant when other QTLs were taken into account. Therefore, the multiple-QTL model is an extension of the single QTL model:


$$y_{ljki}=m+e_{lj}+eb_{ljk}+\sum^{}q_{qi}+\sum^{}qe_{qlji}+x{'}_{i}+x'e_{lji}+r_{ljki}$$

where

q_qi_ was the fixed main effect of the q^th^ QTL in the i^th^ individual

qe_qlji_ was the fixed environment specific q^th^ QTL effect of the i^th^ individual at the lj^th^ environment

*x’_i_* was the background genetic effect of the i^th^ individual with distribution N(0,**G**g) where **G**g was the variance-covariance matrix among total genetic effects given as ***G*** *g* = ***I*** *vg where **I** was the identity matrix of relationships among individuals and vg is the unknown total genetic variance (where * was the Kronecker product)

*x’e_lji_* was the random environment specific background genetic effect of the i^th^ individual at the lj^th^ environment with distribution N(0,**G**ge) where **G**ge was the variance-covariance matrix among environment specific total genetic effects at each of the lj^th^ environments given as ***G*** *ge* = ***I*** * ***V*** *ge* where **I** was the identity matrix of relationships among individuals, and **V**ge was the covariance matrix of environment specific total genetic effects among environments and other terms are identical to the single-QTL model.

### 2.7 Development and analysis of KASP markers

In this section, we aimed to develop and/or analyze KASP markers within QTLs exhibiting the largest QTL×E interactions: QTLs on LGs 1 and 4. SNPs located in the confidence interval (CI) of the QTL on LG1 (47.4-52.3 Mb) were identified through the mapping of ‘Regina’ and ‘Lapins’ RNA-sequencing data (Maldonado et al., 2019; Vimont et al., 2019) and re-sequencing ‘Lapins’ data (Pinosio et al., 2020) on the ‘Regina’ genome sequence (Le Dantec et al., 2020) using the Integrative Genomics Viewers (IGV) software (Thorvaldsdottir et al., 2013) (http://software.broadinstitute.org/software/igv/). Those SNPs, KASP_LG1_50.880 and KASP_LG1_52.362 (named accordingly to their physical position on the ‘Regina’ sweet cherry LG1 in kb, 50.880.246 and 52.362.301 in bp respectively) were then used to develop Kompetitive Allele Specific PCR (KASP) markers, as described in Branchereau et al. (2022). Moreover, markers KASP_9.936 and KASP_9.958 located on LG4 (named accordingly to their physical position on the ‘Regina’ sweet cherry LG4 in kb, 9.935.681 and 9.957.746 in bp, respectively), developed and validated in Branchereau et al. (2022), were used to genotype the population. Allele effect was studied in the five locations separately through analyses of variances in R software, as described in Branchereau et al. (2022). Details concerning the four KASP markers are given in the **supplementary Table S3** (position, sequence, primers, Tm).

## 3 Results

### 3.1 Flowering date evaluation

**Figure 2:**
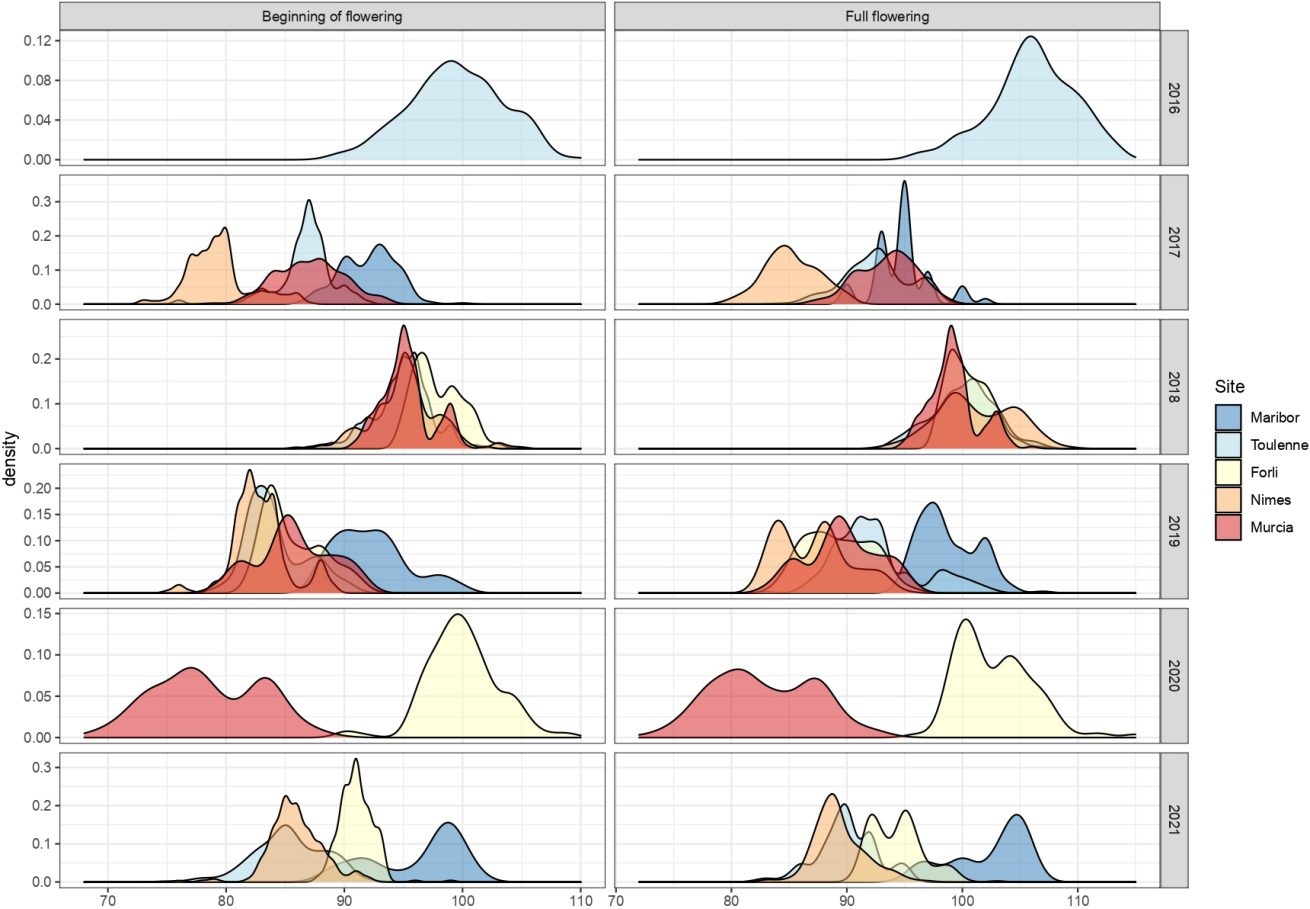
Distribution of beginning of flowering and full flowering (scored in JDs) across the different sites of the MET between 2016 and 2021.

BF and FF were scored across several seasons from 2016 to 2021 in the MET (**Figure 2** and **Table S4**). In 2016, FD was only scored in Toulenne and was late (BF mean = 99.5 JDs) compared to the other years. In 2017, the site where FD occurred the earliest was Nimes (BF=79.4 JDs), followed by Toulenne (BF=86.8 JDs), Murcia (BF = 87.1 JDs) and Maribor (BF=92.1 JDs). In 2018, FD was rather similar in Murcia, Nimes and Toulenne (BF close to 95 JDs), where it started a few days earlier than in Forli (BF=97.9 JDs). In 2019, FD was scored in all sites. It occurred much later in Maribor (BF=92.3 JDs) compared to Nimes, Toulenne, Murcia and Forli (BF from 82.9 to 85.8 JDs). The most extreme FD values across the entire MET were observed in 2020 in Murcia (BF=78.5 JDs) and Forli (BF=100 JDs). Finally, in 2021, FD in Toulenne and Nimes was similar (BF=85.3 and 85.9, respectively) and occurred later in Forli (BF=91.0 JDs) and Maribor (BF=96.1 JDs). Similar observations were made for FF.

Both traits were highly correlated in all environments, with correlation coefficients ranging from 0.79 in Maribor in 2021 to 0.99 in Murcia in 2020 (**Table S5**). Moreover, for each trait, correlations between years in each site were high. Correlations between years were, in average, equal to 0.73 for BF and 0.72 for FF in Forli, 0.66 for BF and 0.58 FF in Maribor, 0.54 for BF and 0.58 for FF in Murcia, 0.74 for BF and 0.71 for FF in Nimes and 0.80 in Toulenne for both PF and FF (**Tables S6**, **S7**). For each year, average correlations between sites were 0.56 for BF and 0.50 for FF in 2017, 0.61 for BF and FF in 2018, 0.49 for BF and 0.55 for FF in 2019, 0.54 for BF and 0.53 for FF in 2020 and 0.60 for BF and 0.56 for FF in 2021 (**Tables S6**, **S7**).

Broad-sense heritabilities (H^2^) for BF were equal to 0.91, 0.90, 0.86, 0.90 and 0.96 in Forli, Maribor, Murcia, Nimes and Toulenne, respectively. For FF, H^2^ were equal to 0.92, 0.88, 0.85, 0.89 and 0.95 in Forli, Maribor, Murcia, Nimes and Toulenne, respectively. The MET broad-sense heritability was equal to 0.96 for both traits.

### 3.2 G×E in the R×L population

**Figure 3:**
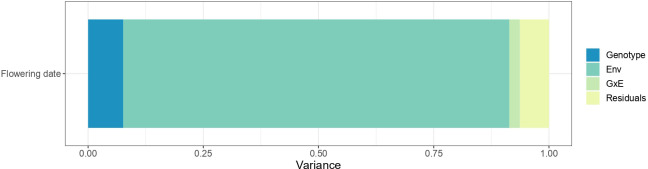
Variance of the fixed effect of environment and the random effects of the genotype, the genotype-by-environment interaction and residuals in flowering date (beginning of flowering).

**Figure 4:**
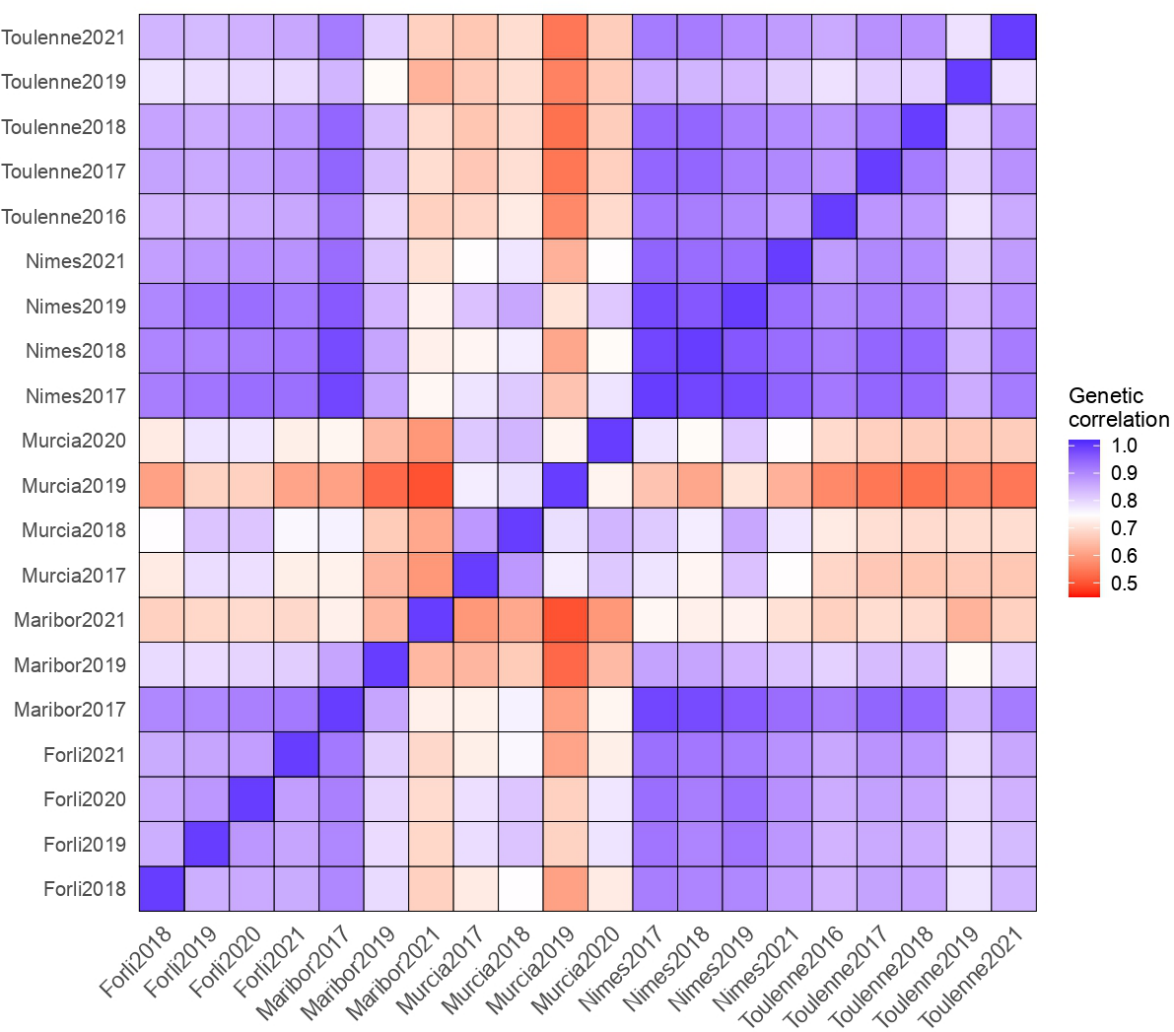
Matrix of pair-wise genetic correlations for beginning of flowering (BF).

The genotype, environment, and G×E effects explained 7.6%, 83.8% and 2.3% of the variance, respectively (**Figure 3**). The high proportion of variation among environmental means in **Figure 3** is supported by highly significant (p< 2.2e-16) differences among the mean effect of each environment. Significant G×E interactions were observed. Estimates of pair-wise genetic correlations between environments for BF ranged from 0.50 to 0.99 and averaged 0.80 (**Figure 4** and **Table S8**). Genetic correlations between seasons within each location were high (0.87 in Forli, 0.74 in Maribor, 0.80 in Murcia, 0.96 in Nimes and 0.85 in Toulenne, on average). Very strong correlations (i.e., higher than 0.80) were observed between environments in Forli, Maribor (except Maribor 2021), Nimes and Toulenne. Correlations between environments in Murcia and other locations were between 0.50 and 0.86 and averaged 0.69. The lowest correlations were found between Murcia and Toulenne (from 0.54 to 0.72, 0.65 in average), and Murcia and Maribor (from 0.50 to 0.76, 0.63 in average).

**Figure 5:**
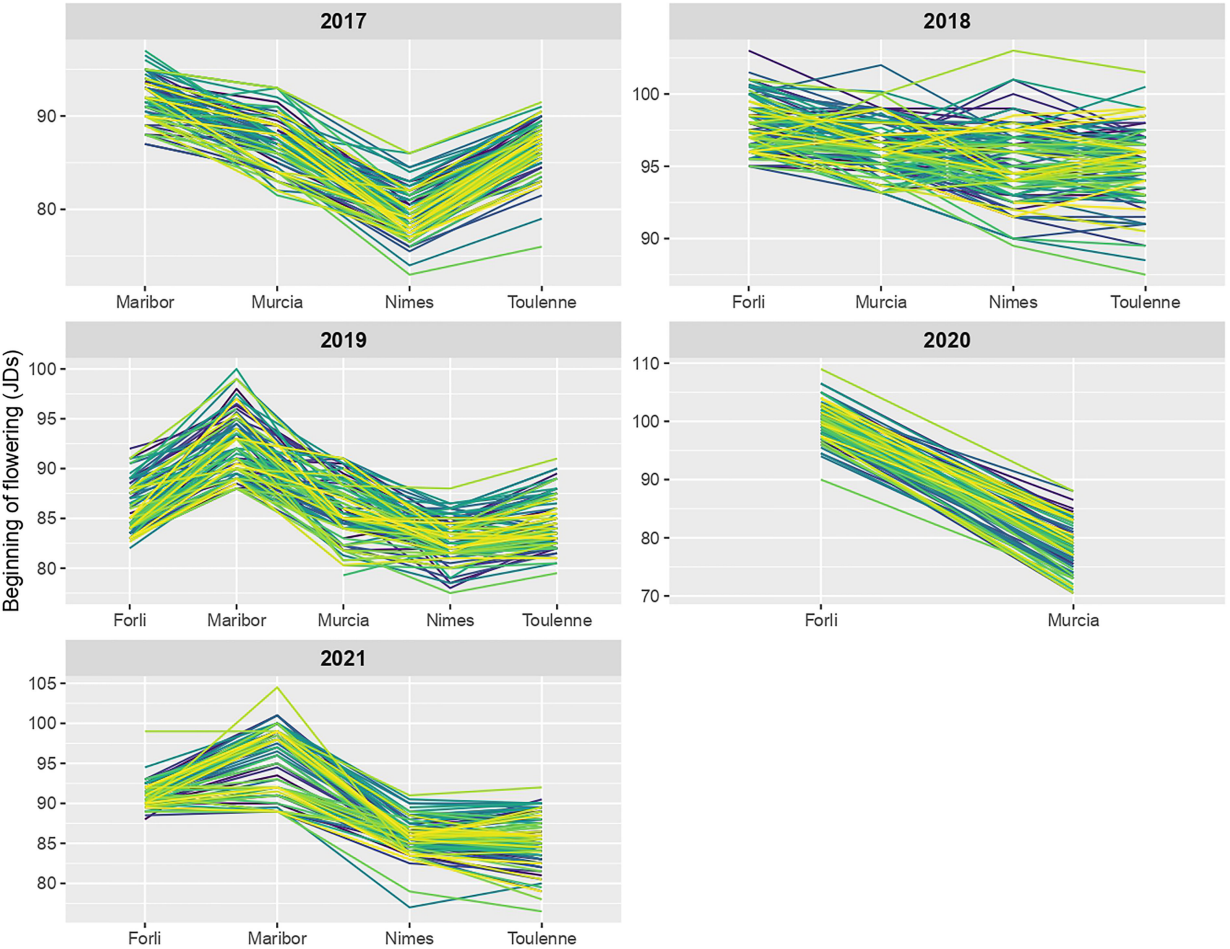
Reaction norms for beginning of flowering (BF) across locations in each year of study. Norm of reaction of each R×L hybrid is shown by a colored line.

For every year of study, reaction norms (**Figure 5**) were not parallel (i.e. different slopes), meaning that genotypes were ranked in different orders in different locations. Genotypes responded differently to various locations, especially in 2018 and 2019. To a lesser extent, changes in individuals ranking were also observed in the five locations across different years (**Figure S6**), most importantly in Maribor and Murcia. In summary, these non-parallel reaction norms showed that rank-change G×E interactions occurred in the MET, and that genotype × location interactions were stronger than genotype × year interactions.

### 3.3 QTL analyses for flowering date

This section will be divided into two parts. In the first one, we will present QTL analyses conducted for BF and FF in each location separately, with single-year and multi-year approaches. In the second part, results from a multi-location – multi-year QTL analysis will be detailed.

#### 3.3.1 QTL detection in each location

QTLs detected for BF with multi-year and single year analyses in each location are presented in **Table 1** (only mean values of PVE and d values across years for each QTL are presented) and **Table S9**, respectively. QTLs detected for FF are presented in **Table S10**.

**Table 1:** Beginning of flowering (BF) QTLs detected with the multi-year analysis in each location.

Location	LG	L (cM)	CI 95% (cM)	Physical position (Mb)	LOD	PVE mean (%)	d mean
Forli	R4	29.6	28.1-31.0	8.35-11.39	24.8	19.1	1.9
	R5	8.2	0.0-34.3	3.30-15.27	7.3	5.3	0.8
	L1	127.5	84.8-152.2	35.68-52.25	10.6	8.75	1.3
	L6	28.8	0.0-86.2	5.79-28.19	6.9	6.65	1.1
Maribor	R4	28.5	24.5-32.6	8.35-17.60	34.6	39.4	3.8
	R5	20.4	0.0-57.5	3.30-20.74	5.7	4.8	0.2
	L4	49.9	33.4-62.8	11.96-21.06	5.8	8.0	1.5
Murcia	R1	47.7	5.7-89.7	0.48-32.09	8.0	7.1	-1.5
	R7	67.0	60.5-70.4	26.43-27.56	9.7	8.6	-1.6
	L1	150.5	148.0-152.2	47.43-52.25	21.7	19.8	2.5
	L6	50.0	5.1-94.8	5.79-28.19	6.3	5.3	1.0
	L7	50.2	34.3-66.1	19.98-26.49	5.9	4.2	-1.1
Nimes	R4	29.6	28.2-30.9	8.35-11.39	32.2	21.5	2.0
	R5	1.0	0.0-7.6	3.30-9.50	10.8	6.4	1.1
	R8	42.9	28.4-55.0	15.90-25.82	6.4	4.0	0.7
	L5	54.2	50.2-55.7	14.90-20.44	9.9	7.6	-1.2
	L6	16.4	0.0-59.3	5.79-18.53	10.7	10.3	1.4
Toulenne	R2	60.8	21.8-84.0	27.76-38.82	8	4.2	0.9
	R4	29.6	27.6-31.6	8.35-11.39	34.9	20.5	2.4
	R5	56.5	46.0-57.5	18.65-20.74	10.3	6.1	-1.3
	L1	144.4	117.3-152.2	40.42-52.25	10.6	7.1	1.4
	L5	54.8	43.6-55.7	14.90-20.44	7.4	4.4	-1.0
	L6	32.7	0.0-99.4	5.79-28.19	16.9	14.3	2.0

LG, linkage group; L, distance from the beginning of the chromosome to the point of maximum LOD in the interval; CI, confidence interval; Physical position of flanking markers on ‘Regina’ v1 genome sequence in mega base pairs (Mb); LOD, logarithm of the odds ratio; PVE mean, mean value of PVE (phenotypic variance explained by the QTL in percentage of the total variation) within separate locations over several years in the multi-environment analysis; d mean, mean value of d (difference X(A) – X(B) according to the environment of evaluation, where A and B are the two homozygotes at the marker loci) in the multi-environment analysis; (+/-), the sign varies according to the environment of evaluation).

For multi-year QTL analysis at Forli, four QTLs were detected for BF on LGs R4 and R5 of ‘Regina’ and LGs L1 and L6 of ‘Lapins’ (**Table 1**). Across single-year analyses for this location, the QTL on LG R4 was the only locus to be significant every season and explained the largest part of the phenotypic variation (**Table S9**). The highest PVE value for the QTL on LG R4 (29.8%) was found in 2018. The QTL on LG L1 was only significant in 2019, where it explained 19.4% of the phenotypic variation. In 2020, an additional QTL was detected on LG L5, explaining 10.8% of the phenotypic variation.

In Maribor, QTLs were detected on LGs R4, R5 and L4. Here again, the QTL on LG R4 was the major QTL, with PVE values ranging from 35% (in 2021) to 41.7% (in 2017) (**Tables 1**, **S9**).

In Murcia, the QTL on LG R4 was not significant. With the multi-year analysis, QTLs were detected on LGs R1, R7, L1, L6 and L7 (**Table 1**). With PVE values ranging from 16.2 to 29.9% in individual years, the QTL on LG L1 showed the largest effect in Murcia (**Table S9**). However, it was not significant in 2019. In 2019, a QTL on LG R3 was detected, explaining 10.9% of the phenotypic variation.

In Nimes, five QTLs were detected with the multi-year analysis, on LGs R4, R5, R8, L5 and L6 (**Table 1**). The QTL on LG R4 was the largest effect QTL, with an average PVE value equal to 21.5% in multi-year analysis, and single-year PVE values ranging from 14.2 to 28.5% (**Table S9**). The QTL on LG L6 was also significant in 2017 and 2021, when it explained 15.2 and 16.6% of the phenotypic variation, respectively.

Finally, in Toulenne, QTLs on LGs R2, R4, R5, L1, L5 and L6 were detected with the multi-year approach (**Table 1**). All these QTLs were detected at least for one year (**Table S9**). Just like in Forli, Maribor and Nimes, the major QTL in Toulenne was the QTL on LG R4, with PVE values up to 31.5% in 2019 (**Table S9**). The QTL on LG L6 was significant in 2016, 2017 and 2018, with PVE values close to 20%, and the QTL on LG L1 was significant in 2021 (PVE = 13.8%). Two additional QTLs were detected in 2019 on LGs R1 and R3.

In summary, the QTL on LG R4 was significant in every single-year or multi-year analysis in all locations except Murcia. In Murcia, the QTL on LG L1 was the major locus in both single-year and multi-year analyses. With multi-year analyses, the QTL on LG L1 was also significant in Forli and Toulenne. QTLs on LGs R1, R7 and L7 were only significant in Murcia, while QTLs on LGs R2, R8 and L4 were only significant in Toulenne, Nimes and Maribor, respectively.

#### 3.3.2 Temperatures in each location and QTL detection

Temperatures in each site for every year of evaluation are described in **supplementary figures S1** for Forli, S2 for Maribor, S3 for Murcia, S4 for Nimes, S5 for Toulenne.

Winter temperatures in 2017-2018 at Forli, (i.e. from December 2017 to the end of March 2018) were the lowest, with a long period of cold occurred in February (**Figure S1**). This year corresponded to the highest estimated PVE value for the QTL on LG R4 (29.8%).

In Maribor, winter temperatures between December and February were lower in 2017 than in 2019 and 2021 (**Figure S2**). The QTL on LG R4 with the highest effect was observed in 2017 with a PVE value reaching 41.7% (**Tables 1**, **S9**). In Murcia, the temperatures during the 2018/2019 winter season were not different from the other years; however, a long period of cold was observed in October/November 2018, as well as in January 2019 (**Figure S3**), the only year in which a QTL on LG R3 was identified.

Temperatures during the month of January in both 2017 and 2021, were particularly low in Nimes (**Figure S4**). For these two years, a QTL on LG L6 was significant.

In Toulenne, years 2016, 2017 and 2018 were relatively different from each other and year 2021 did not show any particular specificity which could be related to the detection of QTL on LG L1 (**Figure S5**).

#### 3.3.3 Multi-location - multi-year QTL analysis

QTLs detected with the twenty environments of the MET with the multi-environment approach are presented in **Table 2** for BF and in **Table S11** for FF. For BF, 12 QTLs were detected, on all LGs of ‘Regina’ and LGs L1, L4, L5 and L6 of ‘Lapins’. Only three of them showed an overall mean PVE higher than 5%: QTLs on LGs R4 (PVE: 20.9%, LOD: 149.6), L1 (PVE: 7.2%, LOD: 39.2) and L6 (PVE: 7.3%, LOD: 42.2). QTL on LG R4 explained in average from 20.1 to 36.1% of the phenotypic variation in Forli, Maribor, Nimes and Toulenne, while it explained only 3.6% in Murcia. A significant negative correlation (-0.75, p = 0.00012) was found between the PVE value of the QTL for BF on LG R4 and the temperature in the 20 environments between October and March (**Figure S7**). The opposite situation was found for the QTL on LG L1. The QTL on LG L1 explained in average 14.8% of the variation in Murcia, and from 3.1 to 7.7% in the other four locations. A correlation coefficient equal to 0.54 (p = 0.013) was found between the PVE value of the QTL on LG L1 and the temperature in the 20 environments (**Figure S8**). Finally, the QTL on LG L6 showed PVE values higher in Nimes (10.2%) and Toulenne (12.4%), compared to the other three locations (from 2.9 to 5.5%). For this QTL, no correlation with temperature data was found (correlation coefficient: -0.056, p = 0.82).

**Table 2:** Beginning of flowering (BF) QTLs detected with the multi-location—multi-year analysis using altogether the twenty environments of the MET.

LG	L (cM)	CI 95% (cM)	Physical position (Mb)	LOD	PVE mean in each location (%)					PVE overall mean (%)	d mean
					Forli (4 years)	Maribor (3 years)	Murcia (4 years)	Nimes (4 years)	Toulenne (5 years)		
R1	27.4	0.0-83.4	0.48-32.09	18.2	2.1	2.6	3.4	2.5	2.3	2.5	-0.6
R2	13.3	0.0-61.3	1.58-32.44	15.2	2.1	1.7	1.8	2.1	1.6	1.9	0.5
R3	47.6	36.2-59.0	12.10-18.91	19.3	1.4	0.6	4.8	2.2	2.5	2.4	0.6
R4	29.4	< 0.5 cM	8.35-11.39	149.6	20.1	36.1	3.6	23.2	24.6	20.9	2.2
R5	1.0	0.0-7.5	3.30-9.50	31.9	4.7	3.8	1.8	7.1	2.3	3.9	0.7
R6	86.0	44.8-104.4	9.77-31.59	16.1	1.9	1.7	5.5	0.9	1.5	2.3	-0.3
R7	60.3	21.8-70.4	1.52-27.56	15.1	1.5	1.2	5.1	0.8	1.6	2.0	-0.5
R8	47.4	46.4-48.4	21.11-25.82	28.3	2.2	1.8	7.0	3.4	3.7	3.7	0.9
L1	145.9	136.9-152.2	47.43-52.25	39.2	7.7	3.1	14.8	3.3	6.2	7.2	1.2
L4	40.5	10.2-62.8	5.00-21.06	16.3	1.5	5.1	2.2	2.9	2.6	2.7	0.4
L5	55.5	< 0.5 cM	14.90-20.44	24.4	4.2	2.0	2.8	5.0	4.0	3.7	-0.8
L6	16.0	8.6-23.4	5.79-8.86	42.2	5.5	2.9	3.2	10.2	12.4	7.3	1.2

LG, linkage group; L, distance from the beginning of the chromosome to the point of maximum LOD in the interval; CI, confidence interval; Physical position of flanking markers on ‘Regina’ v1 genome sequence in mega base pairs (Mb); LOD, logarithm of the odds ratio; PVE (phenotypic variance explained by the QTL in percentage of the total variation) mean in each location, mean value of PVE within separate location over several years in the multi-environment analysis; PVE overall mean, mean value of PVE in the multi-environment analysis; d mean, mean value of d (difference X(A) – X(B) according to the environment of evaluation, where A and B are the two homozygotes at the marker loci) in the multi-environment analysis; (+/), the sign varies according to the environment of evaluation.

For most QTLs, genetic and physical CIs were reduced with multi-location—multi-year analysis. For instance, QTLs on LGs R4 and L5 were detected within a CI of less than 0.5 cM (8.35-11.39 Mb and 14.90-20.44 Mb, respectively). The CI of the QTL on LG L1 (136.9-152.2 cM, 47.43-52.25 Mb) was much reduced compared to the one obtained in multi-year analyses in Forli (84.8-152.2 cM, 35.68-52.25 Mb) and Toulenne (117.3-152.2 cM, 40.42-52.25 Mb), however, it was close to the one found in Murcia (148.0-152.2 cM, 47.43-52.25 Mb).

### 3.4 QTL×E in the R×L progeny

In the multi-environment analysis, QTLs on LGs R3, R4, R5, R7, R8, L1, L5 and L6 were significant for both BF and FF and had the highest LOD values (19.3, 149.6, 31.9, 15.1, 28.3, 39.2, 24.4 and 42.2, respectively) and PVE values (2.4%, 20.9%, 3.9%, 2.0%, 3.7%, 7.2%, 3.7% and 7.3%, respectively), therefore, we decided to study their interactions with the environment (QTL×E). Firstly, single-QTL models were fitted for each QTL.

In contrast to the results from the QTL analyses, QTLs on LGs R5, R8 and L5 were not significant (neither QTL main effect nor QTL×E interaction) for BF in the multi-environment QTL + background linear model (**Table 3**). The most significant QTLs for both main and interaction BF effects were QTLs on LGs R4 and L1. QTLs on LGs L6 and R3 showed significant interactions with environment, while QTL on LG R7 showed a significant main effect.

**Table 3:** Wald tests for fixed effects of single-QTL models and the complete model, for beginning of flowering (BF).

	(a) Single-QTL models				(b) Complete model			
QTL	QTL main effect		QTL × E		QTL main effect		QTL × E	
	SSq	P-value	SSq	P-value	SSq	P-value	SSq	P-value
R3	3	0.376	76	0.048 *	6	0.099	67	0.179
R4	106	< 2.2e-16 ***	135	2.819e-08 ***	116	< 2.2e-16 ***	144	1.628e-09 ***
R5	6	0.123	75	0.057				
R7	8	0.039 *	66	0.2	10	0.022 *	66	0.188
R8	3	0.413	74	0.07				
L1	39	1.858e-08 ***	109	4.553e-05 ***	49	1.512e-10 ***	102	2.322e-04 ***
L5	7	0.079	74	0.051				
L6	7	0.089	86	0.008 **	22	6.909e-05 ***	91	0.003 **

SSq., sum of squares. ***, p<0.001; **, p<0.01; *, p<0.05.

Therefore, we selected QTLs on LGs R3, R4, R7, L1 and L6 and built a complete model combining all these loci. Wald test results are presented in **Table 3** (b). In the complete model, the QTL on LG R3 was not anymore significant. No differences were observed for the QTL on LG R7. QTLs on LGs R4, L1 and L6 remained the most significant loci. For the QTL on LG R4, both QTL main effect and QTL**×**E interaction effect increased in the complete model. Concerning the QTL on LG L1, QTL main effect increased, while the interaction with the environment slightly decreased. Finally, an important increase of the main effect of the QTL on LG L6 was observed in the complete model.

### 3.5 KASP markers in the QTL for flowering date on LG1

Two KASP markers (KASP_LG1_50.880 and KASP_LG1_52.362) have been developed in the CI of the QTL on LG1 and used to genotype the R×L population. Both parental cultivars ‘Regina’ and ‘Lapins’ were heterozygous for these markers; therefore, three genotypes were found in the progeny (**Table 4**).

**Table 4:** Allelic frequency, phenotyping data and statistical analyses for four KASP markers in the R×L progeny.

KASP marker	Genotype	NB ind	Average FD (BF lsmeans) in locations:				
			Forli	Maribor	Murcia	Nimes	Toulenne
KASP markers on LG1							
**KASP_LG1_50.880**	A:A	41	94.0	93.4	87.1	86.2	90.7
	A:T	57	93.6	93.6	87.0	86.0	90.2
	T:T	20	92.6	92.6	85.4	85.1	89.1
	A:A - A:T^a^		+ 0.4 days	- 0.2 days	+ 0.1 days	+ 0.2 days	+ 0.5 days
	P-value		0.044 *	0.775	0.776	0.323	0.062
	A:A - T:T^a^		+ 1.4 days	+ 0.8 days	+ 1.7 days	+ 1.1 days	+ 1.6 days
	P-value		1.86e-06 ***	0.106	2.66e-05 ***	2.46e-04 ***	1.24e-06 ***
	A:T - T:T^a^		+ 1.0 days	+ 1.0 days	+ 1.6 days	+ 0.9 days	+ 1.1 days
	P-value		5.83e-04 ***	0.055	3.12e-05 ***	0.002 **	2.74e-04 ***
**KASP_LG1_52.362**	A:A	38	94.0	94.1	87.9	86.2	90.7
	A:G	63	93.5	93.0	86.5	85.8	90.0
	G:G	17	92.8	93.0	85.2	85.7	89.5
	A:A - A:G^a^		+ 0.5 days	+ 0.9 days	+ 1.4 days	+ 0.4 days	+ 0.7 days
	P-value		0.051	0.012 *	6.53e-06 ***	0.060	0.005 **
	A:A - G:G^a^		+ 1.2 days	+ 0.9 days	+ 2.7 days	+ 0.5 days	+ 1.2 days
	P-value		2.56e-04 ***	0.105	1.44e-09 ***	0.100	7.48e-04 ***
	A:G - G:G^a^		+ 0.7 days	+ 0.9 days	+ 1.3 days	+ 0.2 days	+ 0.5 days
	P-value		0.014 *	0.959	0.001 **	0.718	0.134
KASP markers on LG4							
**KASP_9.936**	A:A	57	92.7	91.6	86.5	84.9	89.0
(Branchereau et al., 2022)	G:A	60	94.4	95.1	87.2	86.8	91.4
	G:A – A:A^a^		+ 1.7 days	+ 3.5 days	+ 0.7 days	+ 1.9 days	+ 2.4 days
	P-value		< 2.2e-16 ***	< 2.2e-16 ***	0.017 *	< 2.2e-16 ***	< 2.2e-16 ***
**KASP_9.958**	C:C	57	92.6	91.4	86.4	84.9	88.9
(Branchereau et al., 2022)	T:C	61	94.4	95.1	87.1	86.9	91.4
	T:C – C:C^a^		+ 1.8 days	+ 3.7 days	+ 0.7 days	+ 2.0 days	+ 2.5 days
	P-value		< 2.2e-16 ***	< 2.2e-16 ***	0.016 *	< 2.2e-16 ***	< 2.2e-16 ***

NB ind, number of individuals; FD, flowering date; BF, beginning of flowering.

a , differences in average flowering dates (lsmeans) between individuals with two different genotypes. ***, p<0.001; **, p<0.01; *, p<0.05.

For KASP_LG1_50.880, significant phenotypic differences between genotypes were found in all locations expect Maribor. In Forli, Murcia, Nimes and Toulenne, the largest BF differences were observed between hybrids with both homozygous genotypes (A:A and T:T) and between hybrids with A:T and T:T genotypes. No significant differences were observed between hybrids with A:A and A:T genotypes, except in Forli. Individuals with either A:A or A:T genotypes were flowering later than individuals with T:T genotype. The allelic effect at this marker was the highest in Murcia (up to 1.7 days of difference between A:A and T:T).

For KASP_ LG1_52.362, significant BF differences between hybrids with the three types of genotypes were found in Forli, Murcia and Toulenne. Murcia was the only location where significant differences were found between all three allelic classes. Homozygous A:A individuals were flowering 1.2, 2.7 and 1.2 days later than homozygous G:G individuals in Forli, Murcia and Toulenne, respectively.

The population was also genotyped with two KASP markers located within the QTL on LG4 (Branchereau et al., 2022). For both markers, only two genotypic classes were found in the population. In Forli, Maribor, Nimes and Toulenne, heterozygous individuals were flowering much later than homozygous individuals (p-values< 2.2e-16). The largest differences were found in Maribor: 3.5 days for KASP_9.936 and 3.7 days for KASP_9.958. In Forli, Nimes and Toulenne, differences were from 1.7 to 2.4 days for KASP_9.936 and from 1.8 to 2.5 days for KASP_9.958. On the other hand, in Murcia, very small significant differences were found (0.7 days for both markers).

## 4 Discussion

### 4.1 Flowering date in the MET

To our knowledge, this study is the first in sweet cherry to report a MET with twenty unique location × year environments. This study shows that FD is highly dependent on the environment, and therefore is not stable across years and locations. Indeed, FD varies between years in a same location as well as between locations for a same year. An interesting observation was made in Nimes in 2017, when FD was very early. Low temperatures scored between December 2016 and the end of January 2017 may have led to a full satisfaction of the CRs, and an important increase of the temperature in February 2017 may have induced an early satisfaction of the HRs. This is a good example of climatic conditions that can induce important advances in FD, which can have dramatic consequences if the temperatures decrease again (spring frost damages) (Wenden et al., 2017). Correlations between years within a location were higher than correlations between locations. Nevertheless, estimates of broad-sense heritability were high, even at the whole MET level, in the same range as those estimated in prior studies in sweet cherry and other *Prunus* species (Dirlewanger et al., 2012; Castède et al., 2014; Cai et al., 2018; Calle et al., 2020; Branchereau et al., 2022). Castède et al. (2014) estimated similar heritabilities using the same R×L population planted on own roots and evaluated at Toulenne, suggesting that grafting did not have any impact on heritability in our study. Heritability values for both FD stages were higher in Toulenne than in other locations. This can be explained by a larger number of years of measurements available in this location.

The G×E linear mixed model revealed strong environment and interaction effects, and the estimation of pair-wise genetic correlations between environments suggested genotypes ranking modifications across environments, especially in Murcia. This was confirmed by non-parallel reaction norms, highlighting significant rank-change G×E interactions in our MET.

Jung et al. (2020) found a significant effect of G×E on floral emergence (equivalent to beginning of flowering) in apple, and highlighted as well the strong effect of the environment on this trait. The results we obtained for FD contrast with the limited effect of G×E on maturity date previously reported in sweet cherry (Hardner et al., 2019). In Hardner et al. (2019), genetic correlations for maturity date were much higher (from 0.82 to 1.0, averaged 0.95) than the one we calculated for FD (from 0.50 to 0.99, averaged 0.80). However, different environments were studied, and the environmental and climatic range has a strong impact on the estimation of G×E. Moreover, FD might be more dependent on the environment than maturity date. Indeed, FD depends on dormancy release which is closely related to temperature to fulfill CRs and HRs, and therefore requires chill accumulation followed by heat accumulation, while maturity date may be primarily dependent on heat accumulation after flowering (Gucci et al., 1991).

### 4.2 Major FD QTL on LGs 1 of ‘Lapins’ and 4 of ‘Regina’

In all locations, few QTLs were detected with the single year analyses. Multi-year analyses improved detections: more QTLs were detected when combining several years of phenotypic data, and CIs were reduced. Using the 20 environments altogether further increased the power and the accuracy of the QTL detection. Many loci accounting for a very small proportion of the phenotypic variation were significant in the multi-location—multi-year analysis. Multi-environment analysis allowed to detect a much higher number of QTLs than multi-year analyses in separate locations. For some loci (e.g. QTLs on LGs R4 and L5), the genetic position of the CI (in cM) was reduced. However, due to the low marker density of the genetic maps, it did not improve the physical position. Overall, the large number of QTLs detected confirmed the complex polygenic control of FD (Dirlewanger et al., 2012; Castède et al., 2014; Branchereau et al., 2022). Only three loci explained more than 5% of the phenotypic variation.

Castède et al. (2014) studied the same R×L population planted on own roots in Toulenne and detected QTLs for FF on LGs R4, R5 and L1 in single year analyses. In the study reported here, the QTL on LG R4 was the only one to be significant every year. In the multi-year analysis combining altogether six years of measurements (period 2006-2012), QTLs were found on LGs R4, R5, R8, L1 and L2 (Castède et al., 2014). In our study, we confirmed that the QTL on LG R4 was the most stable QTL in Toulenne, but also in Forli, Maribor and Nimes. The multi-year analysis we performed in Toulenne (five years) for FF led to the detection of QTLs on LGs R2, R4, R5, L1, L5 and L6. Therefore, QTLs in common in both studies were QTLs on LG R4 and L1 (the QTL on LG R5 mapped in different chromosomal regions in both studies). These differences may be due to year, tree age, rootstock or micro-environmental effects, as well as any combination of these factors and confirm the complexity of the genetic determinism of this trait. In Branchereau et al. (2022), QTLs on LGs 1 and 4 were also detected in the ‘Regina’ × ‘Garnet’ population planted in Toulenne. QTL on LG1 was found in ‘Garnet’ cultivar but was not stable across years, likewise the QTL on LG1 in ‘Lapins’ cultivar in Toulenne. On the other hand, the QTL on LG4 of ‘Regina’ was very stable, detected every year of study (i.e. over ten years) (Branchereau et al., 2022), as observed in this study in Forli, Maribor, Nimes and Toulenne. This QTL was detected in a physical CI of the same range in both studies.

Conducting QTL mapping in each location separately and then comparing the results allowed us to discover that the QTL on LG R4 was found in all locations except Murcia, and that, in this location, the major QTL was located on LG L1. The QTL on LG L1 was also detected across some years in Forli and Toulenne. This LG1 QTL has been identified in sweet cherry cultivars ‘Cristobalina’, ‘Garnet’ and ‘Lapins’ in the chromosomal region of the *DAM* genes (Dirlewanger et al., 2012; Castède et al., 2014; Calle et al., 2020; Branchereau et al., 2022). ‘Cristobalina’ is an extra-early blooming cultivar with very low CRs, and a recent study revealed that it carries structural mutations in the *DAM* genes region that might be responsible of this phenotype (Calle et al., 2021). Therefore, the QTL on LG1, covering *DAM* genes, seems to be related to the CRs. Nevertheless, the situation is probably highly complex. Indeed, Castède et al. (2014) reported CR QTLs on LGs R1 and G1 by working with a population derived from the cross between ‘Regina’ and ‘Garnet’ but the corresponding peaks mapped in a clearly different genomic position as the one carrying the *DAM* genes. The same result was observed a few years later by conducting the same type of analysis on population ‘Regina’ × ‘Lapins’, in which CR QTLs were detected on LG L1 but again, in an upstream chromosomal region (Quero-Garcia, comm. pers.). On the other hand, the LG4 QTL might be more related to HRs but also to CRs since Castède et al. (2014) found clear co-localizations of bloom date, CR and HR QTLs on LG R4. We calculated significant positive and negative correlations between the temperature and the proportion of the phenotypic variance explained by QTLs on LGs L1 and R4, respectively. This suggests that the QTL on LG L1 plays a major role in warm region environments (where HRs are easily fulfilled but not CRs), while QTL on LG R4 is more significant in colder regions (where CRs are easily fulfilled but not HRs). This result might contribute to future experimental designs aimed at elucidating the complex regulation of genes involved in FD, underlying these QTL regions, in particular its interaction with temperature.

In our MET, QTLs on LGs 1 and 4 may be described as ‘conditionally neutral’ QTLs, because they are detected in only specific environments (El-Soda et al., 2014). If a QTL is detected in some environments but not in others, it implies QTL×E interactions.

### 4.3 QTL×E interactions and MAS

In both single-QTL and multiple-QTL models, loci with most significant and largest QTL×E interactions were QTLs on LGs R4 and L1. This is in accordance with the QTL detections that revealed that both loci were large-effect environment-specific QTLs.

In a context of MAS, both QTL main effect and QTL×E effect should help in the selection of hybrids particularly adapted to specific environments. For instance, a breeder aiming at the release of new cultivars for cold production areas, will put more weight on the QTL of LG4, if using cultivar ‘Regina’ as a parent, by selecting ‘late flowering’ alleles in order to avoid the risk of frost damages. On the opposite, a cultivar adapted to regions characterized by warm winters with a lack of chill, which will derive from ‘Lapins’, will need to inherit ‘early flowering’ alleles of LG1 QTL, most likely associated to low CRs. Finally, for intermediate environments such as the ones represented in our study by the sites of Toulenne, Forli or Nimes, it might be advisable to combine ‘early flowering’ alleles from the LG1 QTL of ‘Lapins’ with ‘late flowering’ alleles of the LG4 QTL of ‘Regina’, by trying to combine in a single hybrid ‘sufficiently’ low CRs with ‘sufficiently’ high HRs.

These hypotheses/strategies were supported by the analysis of four KASP markers located within the QTLs on LGs 1 and 4. Indeed, the effect of the LG1 KASP markers was larger in Murcia (the warmest environment of the MET) than in other locations, while LG4 KASP markers (Branchereau et al., 2022) played a major role in Maribor, Toulenne, Nimes and Forli. Additionally, two markers developed by Calle et al. (2021) and located within the *DAM* genes on the LG1 might be useful. Both markers were developed from the extra-early cultivar ‘Cristobalina’ for the detection of structural mutations (within the *DAM* genes region) associated to early FD and low CRs in sweet cherry (Calle et al., 2021). More recently, in peach, KASP markers were developed in the region spanning from 43.58 to 43.78 Mb on the chromosome 1 (Pp01) and validated to predict CRs (Demirel et al., 2023). These markers, located near the *DAM* genes, are located 1 Mb upstream from those that we developed within the LG1 QTL (orthologous positions of KASP_LG1_50.880 and KASP_LG1_52.362 on the peach chromosome 1 are 44.60 Mb and 45.78 Mb, respectively). However, the strong association we found for KASP_LG1_50.880 and KASP_LG1_52.362 with FD in Murcia shows that these markers could be useful for selection in warm environments.

Therefore, all these markers should contribute to establish a complete MAS strategy for FD, their choice depending of the climatic conditions of the place where cherry trees will be planted. This study demonstrates that MAS should be performed with different markers if the climate is warm or cold to select well adapted genotypes to a specific region. In climates with warm winters, genotypes with alleles responsible of early flowering at markers on LG1 should be selected, while in climates with cold winter, genotypes with alleles responsible of late flowering on LG4 should be selected to avoid frost damage. In intermediate environments, selection should be done on both loci by screening genotypes with ‘early flowering’ alleles on LG1 and ‘late flowering’ alleles on LG4.

## 5 Conclusion

To our knowledge, this study is the first in sweet cherry to perform QTL analyses in a complex MET and estimating G×E and QTL×E interactions for FD. We showed that FD is highly dependent on the environment with important inter-annual and inter-location variations. Differences of individuals ranking between environments were the major source of G×E detected in this study. QTL×E plays a major role in adaptation to environment changes. Our study revealed that two major FD loci in sweet cherry, located on LGs 1 and 4, exhibited strong QTL×E. Therefore, this study provides relevant information for the choice of stable QTLs in specific environments in order to target them in MAS. Molecular markers have been developed in both loci, and therefore could be used simultaneously to start a complete MAS strategy for FD and develop new cultivars well adapted to their cultivation area. Molecular breeding based on these markers could be undertaken to select genotypes for specific climatic conditions. This study focused on FD, a key trait in sweet cherry breeding, but other traits related to fruit quality could be studied as well. Therefore, this unique sweet cherry MET paves the way for molecular breeding strategies.

## Data availability statement

The datasets presented in this study can be found in online repositories. The names of the repository/repositories and accession number(s) can be found in the article/**Supplementary Material**.

## Author contributions

JQ-G and ED designed the experiments and provided financial support; DA, JP, AI, DG, FB, GL-O, FG-M, and BQ-T carried out or supervised phenotyping; CB analyzed the data; CH and BW made contributions to data analysis; CB and LLD made bioinformatics sequence analysis for KASP design, CB, JQ-G, and ED wrote the manuscript. All authors contributed to the article and approved the submitted version.

## Supplementary material

Supplementary Figure 1 Temperature deviation to the mean in Forli in 2018, 2019, 2020 and 2021 for the period spanning from October to April. The mean was calculated using the temperature data from 2010 to 2021.

Supplementary Figure 2 Temperature deviation to the mean in Maribor in 2017, 2019 and 2021 for the period spanning from October to April. The mean was calculated using the temperature data from 2010 to 2021.

Supplementary Figure 3 Temperature deviation to the mean in Murcia in 2017, 2018, 2019 and 2020 for the period spanning from October to April. The mean was calculated using the temperature data from 2010 to 2021.

Supplementary Figure 4 Temperature deviation to the mean in Nimes in 2017, 2018, 2019 and 2021 for the period spanning from October to April. The mean was calculated using the temperature data from 2010 to 2021.

Supplementary Figure 5 Temperature deviation to the mean in Toulenne in 2016, 2017, 2018, 2019 and 2021 for the period spanning from October to April. The mean was calculated using the temperature data from 2010 to 2021.

Supplementary Figure 6 Reaction norms for Beginning of Flowering (BF) across years in each location of study. Norm of reaction of each R×L hybrid is shown by a line.

Supplementary Figure 7 Representation of the PVE values of QTL on LG R4 obtained with the multi-location—multi-year analysis as a function of the mean temperature for the period from October to February.

Supplementary Figure 8 Representation of the PVE values of QTL on LG L1 obtained with the multi-location—multi-year analysis as a function of the mean temperature for the period from October to February.
