# Supplementary figures and images for: Genotype-by-environment and QTL-by-environment interactions in sweet cherry (Prunus avium L.) for flowering date
Source: Front Plant Sci. 2023 Mar 2;14:1142974. doi: 10.3389/fpls.2023.1142974 (PMC10017975; doi:10.3389/fpls.2023.1142974)

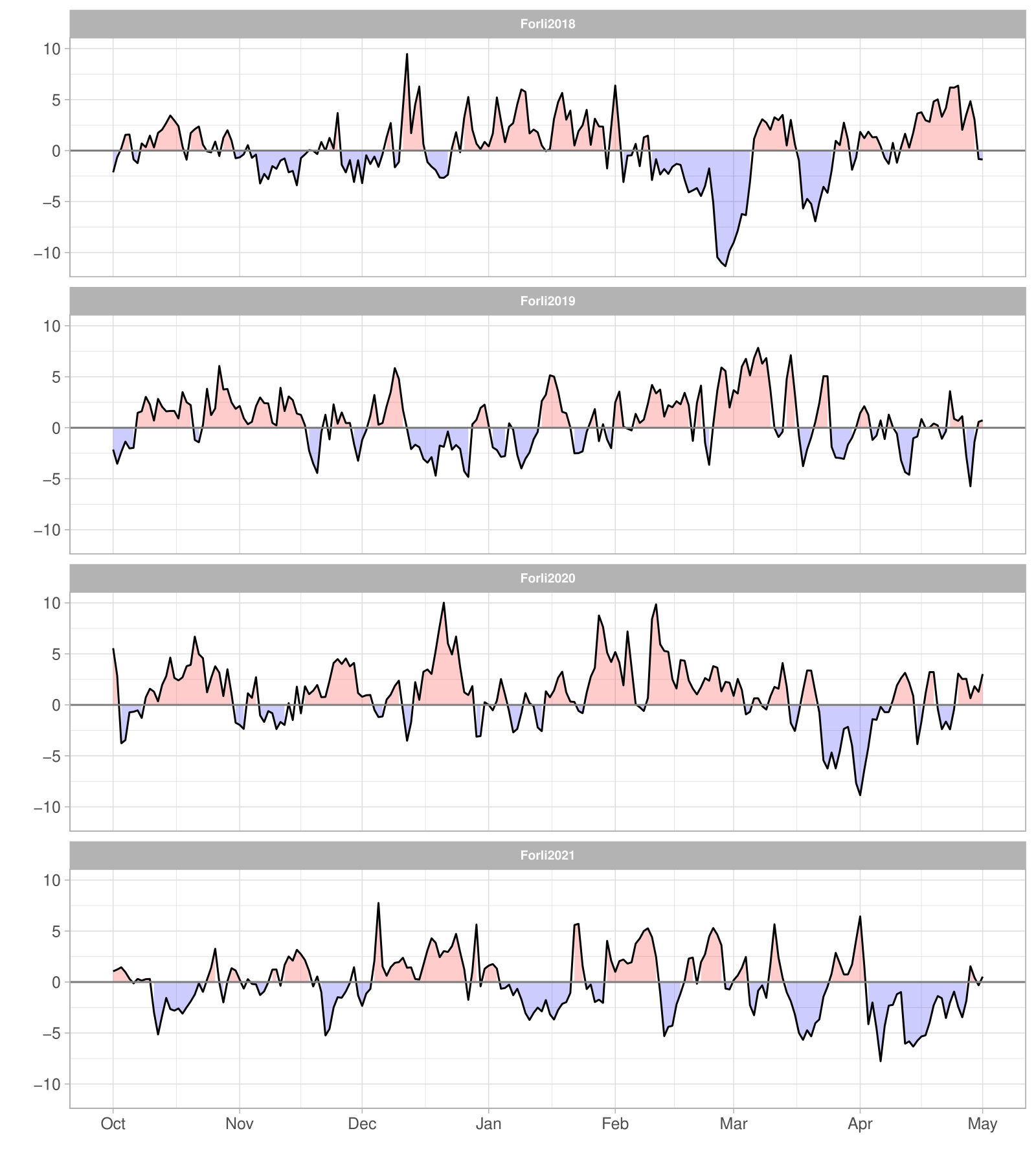

Supplement: Supplementary Figure 1 — Temperature deviation to the mean in Forli in 2018, 2019, 2020 and 2021 for the period spanning from October to April. The mean was calculated using the temperature data from 2010 to 2021. [file Image_1.tiff]

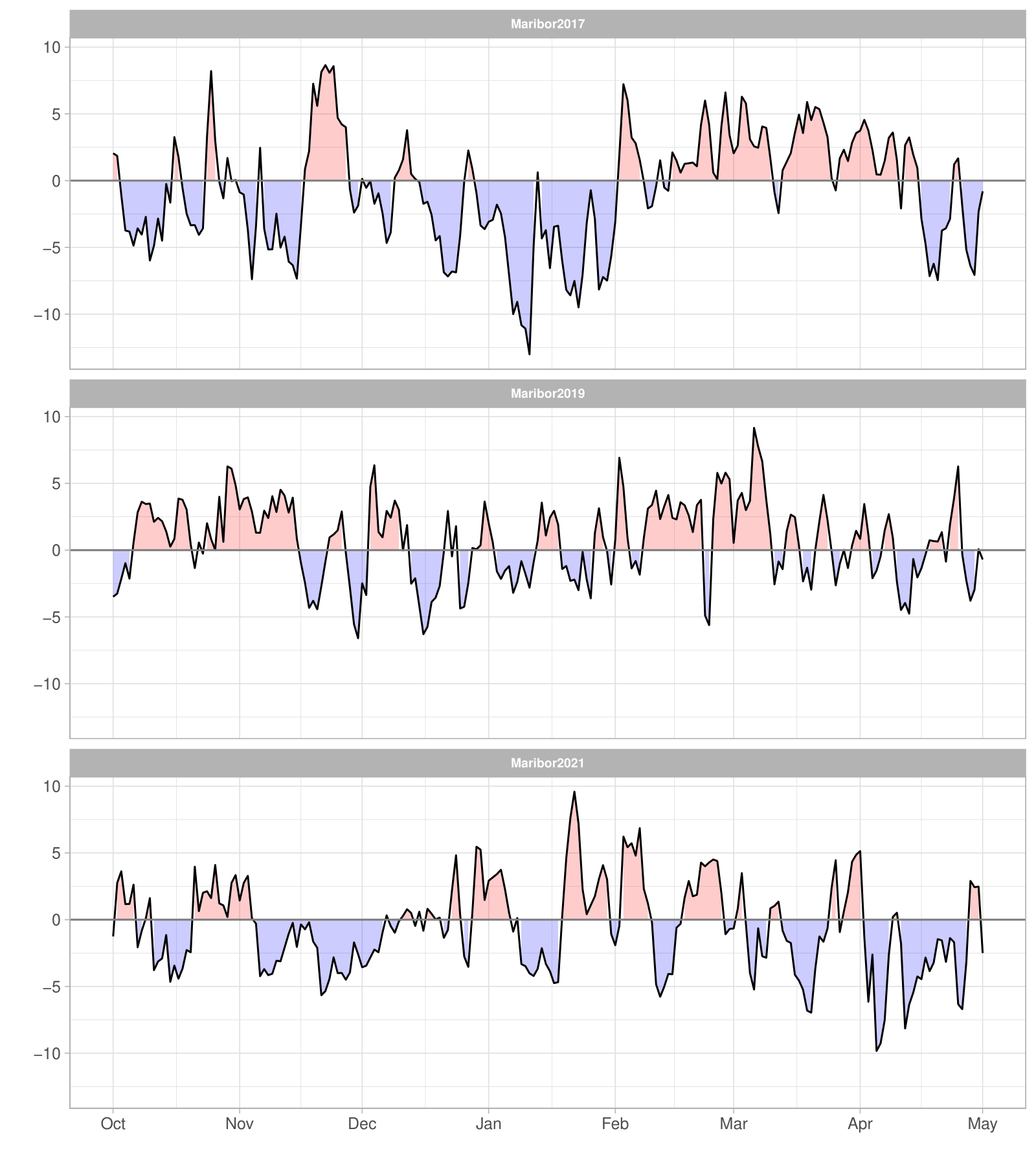

Supplement: Supplementary Figure 2 — Temperature deviation to the mean in Maribor in 2017, 2019 and 2021 for the period spanning from October to April. The mean was calculated using the temperature data from 2010 to 2021. [file Image_2.tiff]

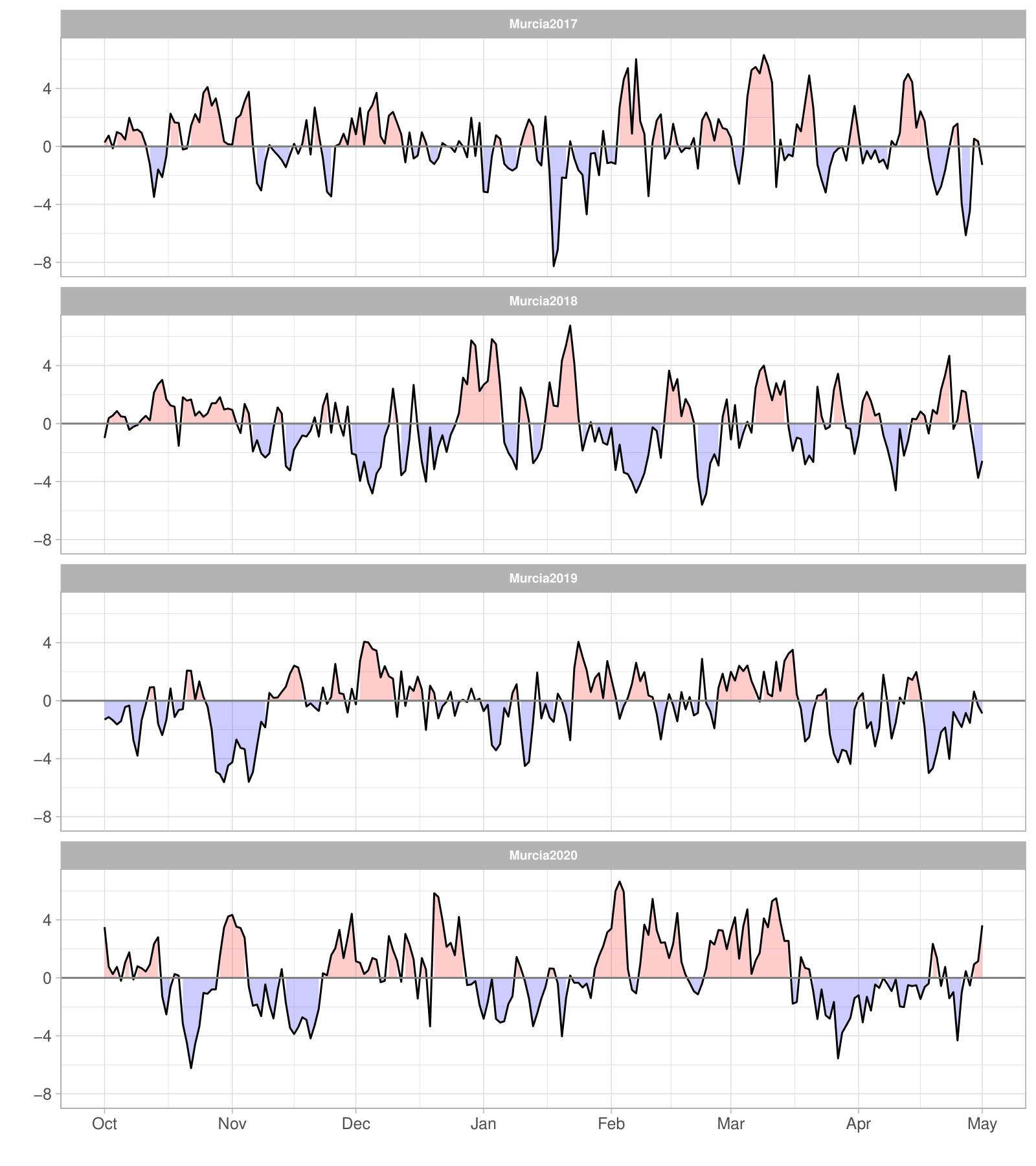

Supplement: Supplementary Figure 3 — Temperature deviation to the mean in Murcia in 2017, 2018, 2019 and 2020 for the period spanning from October to April. The mean was calculated using the temperature data from 2010 to 2021. [file Image_3.tiff]

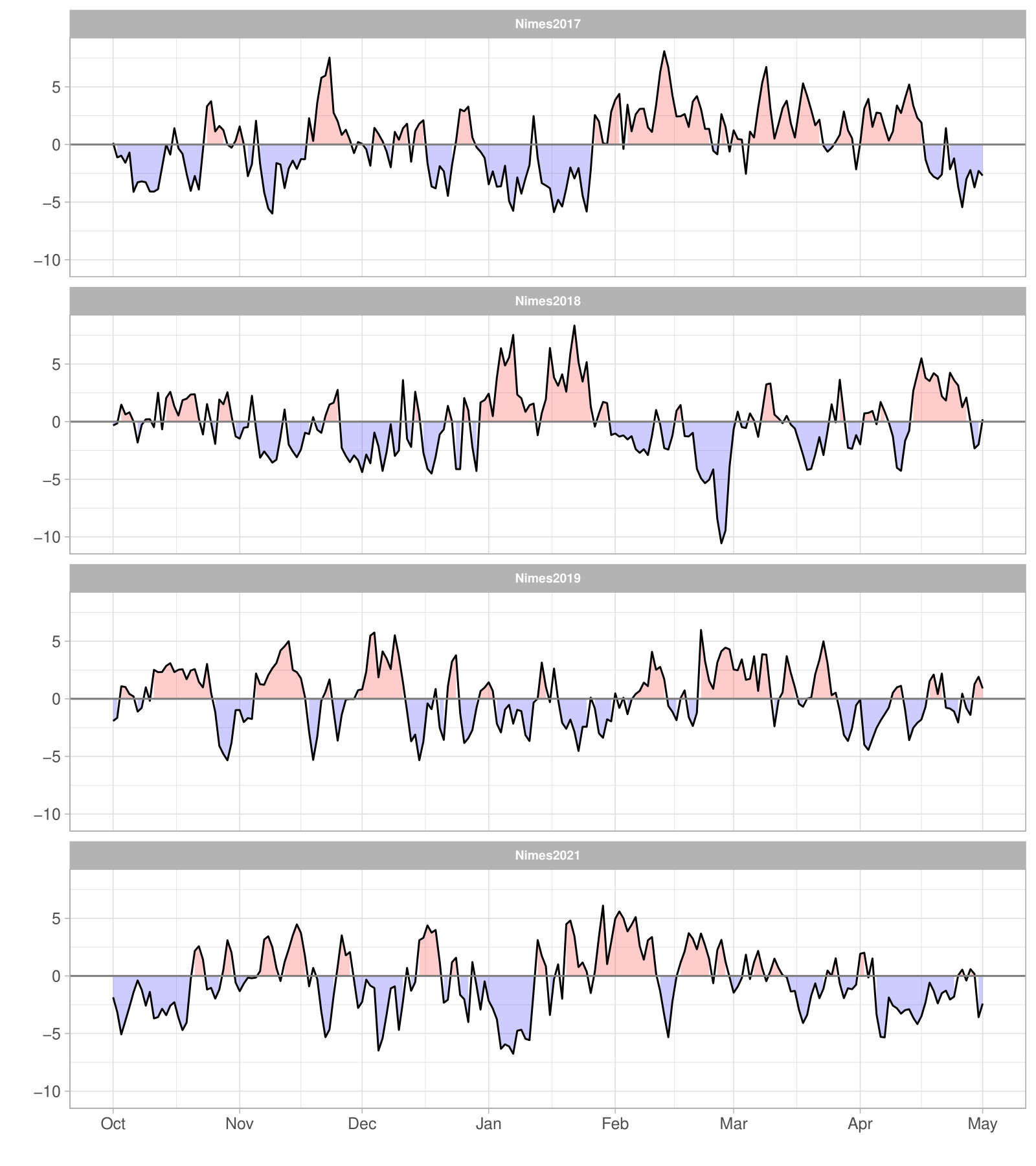

Supplement: Supplementary Figure 4 — Temperature deviation to the mean in Nimes in 2017, 2018, 2019 and 2021 for the period spanning from October to April. The mean was calculated using the temperature data from 2010 to 2021. [file Image_4.tiff]

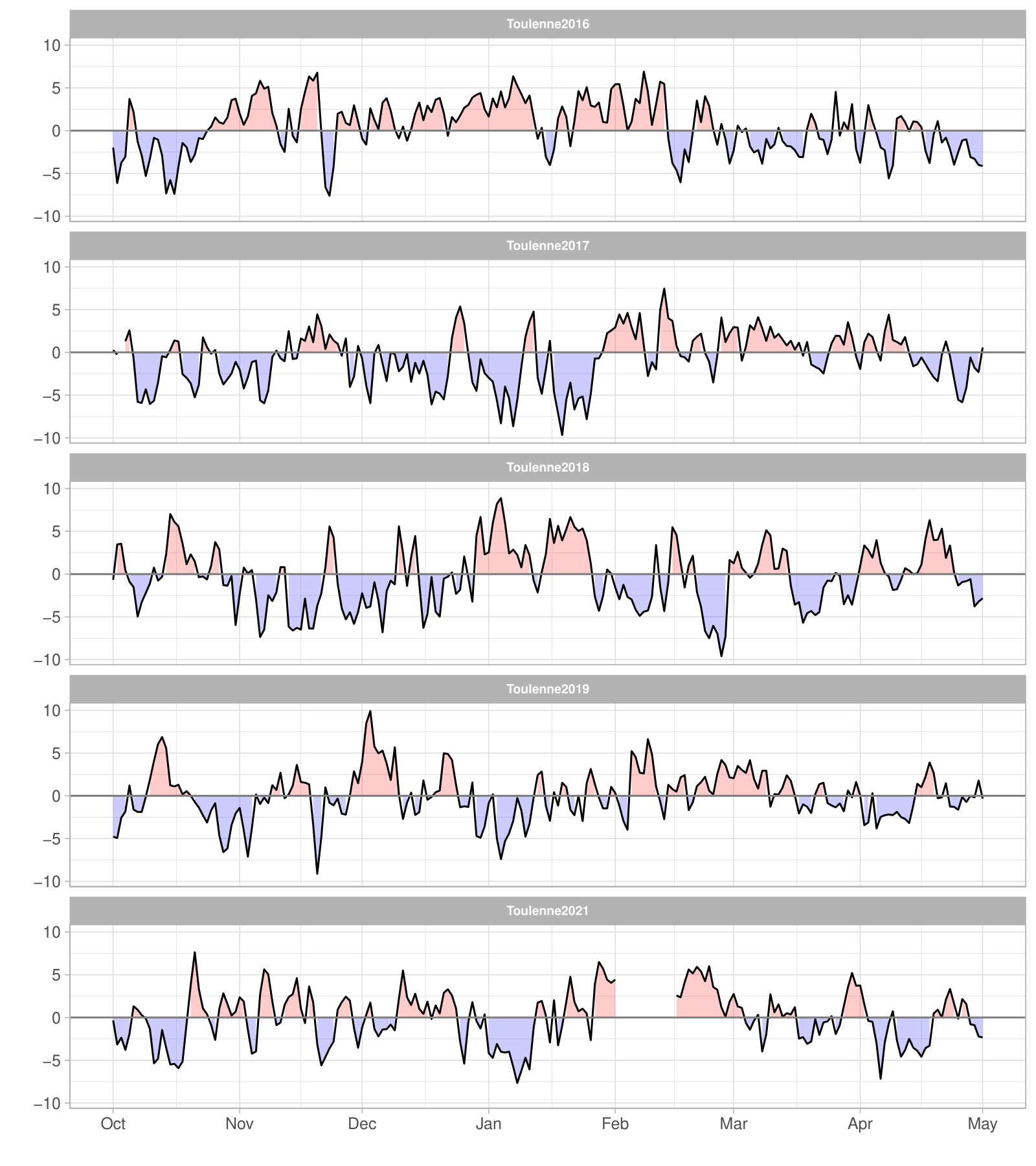

Supplement: Supplementary Figure 5 — Temperature deviation to the mean in Toulenne in 2016, 2017, 2018, 2019 and 2021 for the period spanning from October to April. The mean was calculated using the temperature data from 2010 to 2021. [file Image_5.tiff]

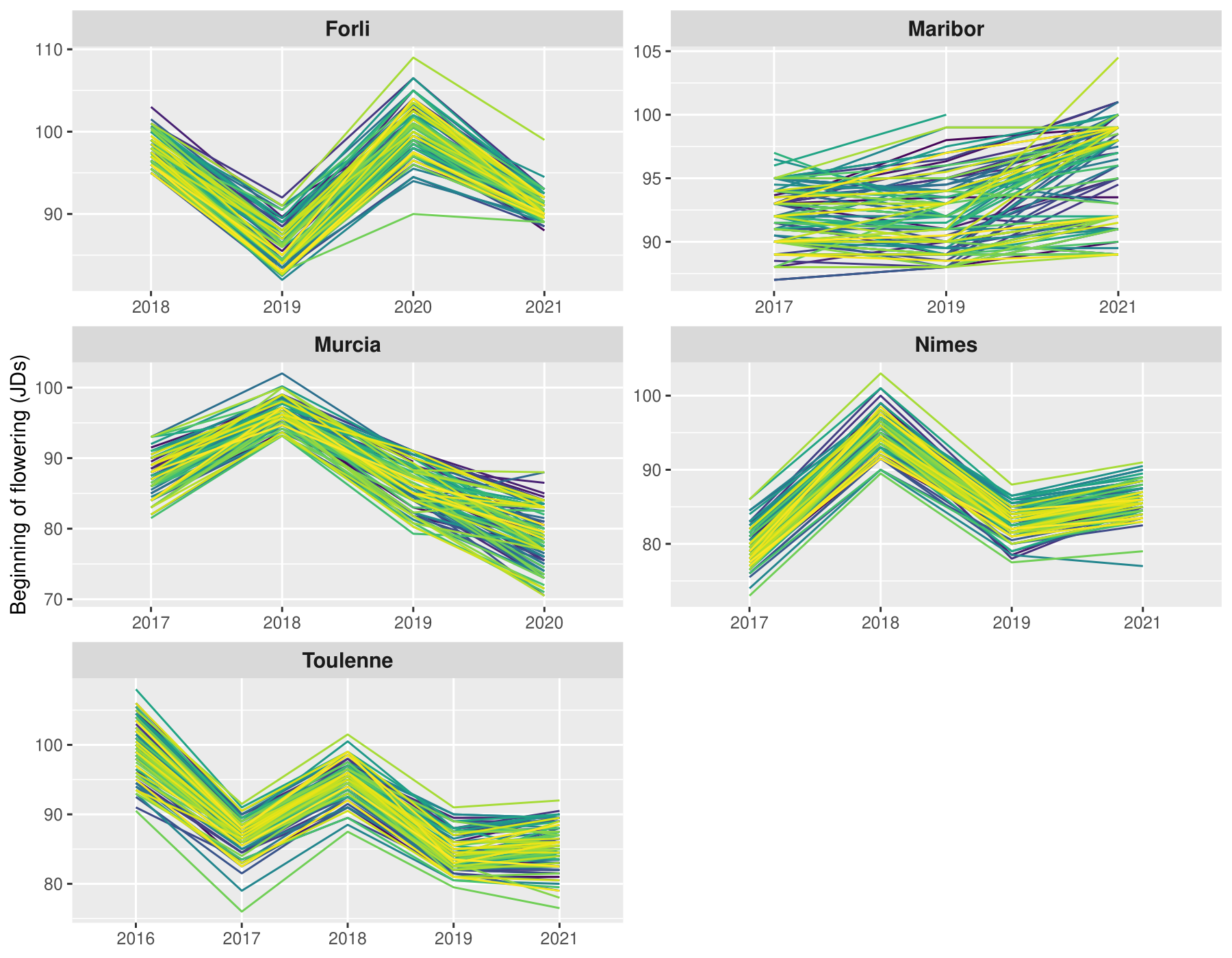

Supplement: Supplementary Figure 6 — Reaction norms for Beginning of Flowering (BF) across years in each location of study. Norm of reaction of each R×L hybrid is shown by a line. [file Image_6.tiff]
